# Keyhole supraorbital eyebrow approach for fully endoscopic resection of tuberculum sellae meningioma

**DOI:** 10.3389/fsurg.2022.971063

**Published:** 2022-09-07

**Authors:** Xialin Zheng, Dongqi Shao, Yu Li, Longjie Cai, Shan Xie, Zhixiang Sun, Zhiquan Jiang

**Affiliations:** ^1^ School of Continuing Education, Anhui Medical University, Hefei, China; ^2^ Department of Neurosurgery, The First Affliated Hospital of Bengbu Medical College, Bengbu, China

**Keywords:** tuberculum sellae meningioma, keyhole supraorbital eyebrow approach, endoscopic resection, transcranial resection, endoscope

## Abstract

**Background:**

The fully endoscopic supraorbital trans-eyebrow keyhole approach is a technique utilized for the transcranial resection of tuberculum sellae meningioma (TSM). Surgery is the first choice for TSM treatment. This study aimed to summarize and analyze the safety, feasibility, limitations, and technical requirements of the fully endoscopic supraorbital trans-eyebrow keyhole approach for TSM resection.

**Methods:**

Data of 19 TSM fully endoscopic supraorbital trans-eyebrow keyhole approach resections cases (six and 13 on the left and right eyebrows, respectively) were retrospectively analyzed at the Neurosurgery Department of the First Affiliated Hospital of Bengbu Medical College (Bengbu, China) from August 2015 to March 2022.

**Results:**

All 19 patients were diagnosed with meningioma (World Health Organization grade I), and according to the scope of tumor resection (EOR), 18 patients (94.7%) had gross total resection (GTR), and one patient (5.3%) had near-total resection (NTR). Preoperative chief complaints were symptomatic visual dysfunction (*n* = 12), headache and dizziness (*n* = 6), and accidental discovery (*n* = 1). Postoperative visual function improved in 83.3% of cases (10/12), and headache and dizziness were relieved in 83.3% of cases (5/6 patients). Postoperative intracranial infection occurred in one case and was cured by external drainage of the lumbar cistern and anti-infective treatment. Two cases of frontal lobe injury were discharged after conservative treatment. There was no postoperative olfactory dysfunction, eyelid ptosis, cerebrospinal fluid leakage, or death. There were no reports of disease recurrence or death during the 3-month follow-up at an outpatient clinic or by telephone.

**Conclusion:**

Fully endoscopic TSM resection through the keyhole approach is safe and feasible. It can be used to explore angles that cannot be seen under a microscope and show the true value of endoscopy technology. The endoscopic equipment and technical skills of the surgeon and surgical team are important in this technique.

## Introduction

Although surgical resection is the first choice for the treatment of tuberculum sellae meningioma (TSM), the degree of tumor resection and visual function pose challenges (1–3). With the improvement of microscopy, the transcranial approach (TCA) (e.g., pteral point and subfrontal approaches) has been established as the standard method for surgical TSM resection. Nevertheless, limitations still exist regardless of transcranial microsurgery type (1). In recent years, the use of the endoscopic endonasal approach (EEA) has become increasingly common owing to the rapid advancement of neuroendoscopy (4). Compared with TCA, the main advantage of EEA is the early treatment of the tumor base and Simpson grade I resection. However, cerebrospinal rhinorrhea, sellar floor reconstruction, anosmia, and nasal symptoms limit further EEA development.

The supraorbital approach has been shown as the most suitable method for TSM treatment, offering the advantages of less trauma and faster recovery compared with other TCAs (5, 6). Some scholars have proposed that there are blind areas in the microscopical approach through the eyebrow arch, and the use of angle neuroendoscopy can effectively solve this problem (7). Berhouma et al. (8) first suggested the feasibility of total endoscopic resection of anterior middle skull base lesions through the keyhole of the eyebrow arch approach. Arnaout et al. (9) performed eight dissections through the supraorbital approach on four cadaver heads using a microscope and an endoscope and compared the visibility and accessibility of the anterior cranial fossa. They concluded that both approaches provide similar visibility and accessibility for the surgeon. Based on this evidence, we evaluated the surgical techniques, clinical efficacy, and technical requirements for the resection of TSM by the supraorbital approach at the First Affiliated Hospital of Bengbu Medical College (Bengbu, China). This case series analysis and suggested algorithm aim to guide neurosurgeons in managing TSMs.

## Methods

### Patient selection

**Figure 1:**
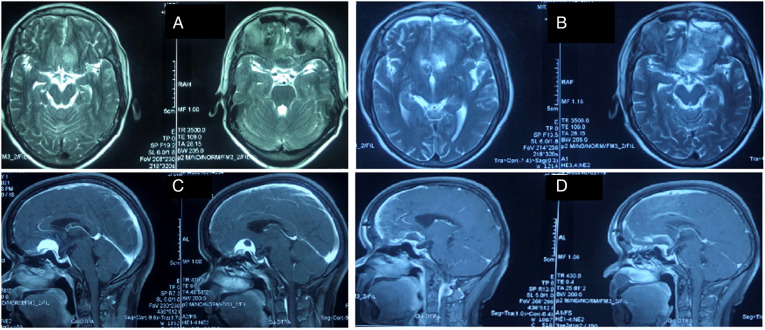
Magnetic resonance imaging of the patients (**A,C**) Preoperative (**B,D**) postoperative.

We reviewed the clinical outcomes and imaging data of 19 patients with TSM who underwent pure neuroendoscopic transeyebrow approach surgery at our hospital from August 2015 to March 2022. The baseline characteristics of patients are shown in Table 1. Computed tomography scan, magnetic resonance imaging (MRI) scan with enhancement, computed tomography angiography (CTA), visual field assessment, and examination of hormone levels in venous blood were preoperatively performed (Figure 1). The postoperative pathological analysis confirmed the presence of World Health Organization (WHO) grade I meningioma. All procedures were performed by a single surgical team. All patients underwent surgery for the first time.

**Table 1:** Characteristics of patients with tuberculum sellae meningioma (TSM).

General information		
female	14 (73.7%)	
age	32–70, 56	
Manifestations	Headache	6 (31.6%)
	Hypopsia	12 (63.2%)
	Physical examination findings	1 (5.3%)
Eyebrow arch approach (Left/Right)	Left	6 (31.6%)
	Right	13 (68.4%)
Mean tumor volume, cm^3^	3.6 ± 2.5	

Patients with meningiomas that did not have tumor epicenter at the tuberculum sella/posterior planum region and had invasive parasellar meningiomas arising from the cavernous sinus and/or Meckel's cave and clinoidal meningiomas were excluded from this analysis. Collected data included demographics, MRI characteristics, preoperative and postoperative clinical status, vision/visual field assessment, and complications. Differences in the skills of surgeons during tumor resection procedures were recorded. All patients were followed up for at least 3 months with MRI analysis and pituitary hormone testing. This study was approved by the Research Ethics Committee of Bengbu Medical College. The need for informed consent was waived due to the retrospective nature of the study. Nevertheless, the confidentiality of patient data was protected according to the tenets of the Declaration of Helsinki.

### Surgical method

**Figure 2:**
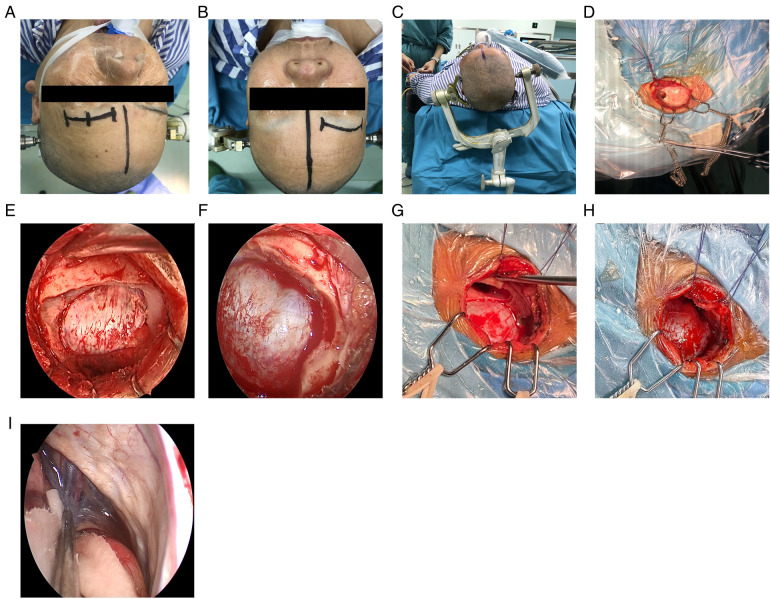
Operating procedure. (**A,B**) Location of the surgical approach. (**C**) The DORO head brace was used to fix the head of the patient. (**D**) Upward retraction using two “fishhook-morphous” retractors. **(E)** Removal of the small free bone flap. (**F**) Supraorbital nerve. (**G,H**) Opening of the frontal sinus: before and after. (**I**) Release of cerebrospinal fluid.

**Figure 3:**
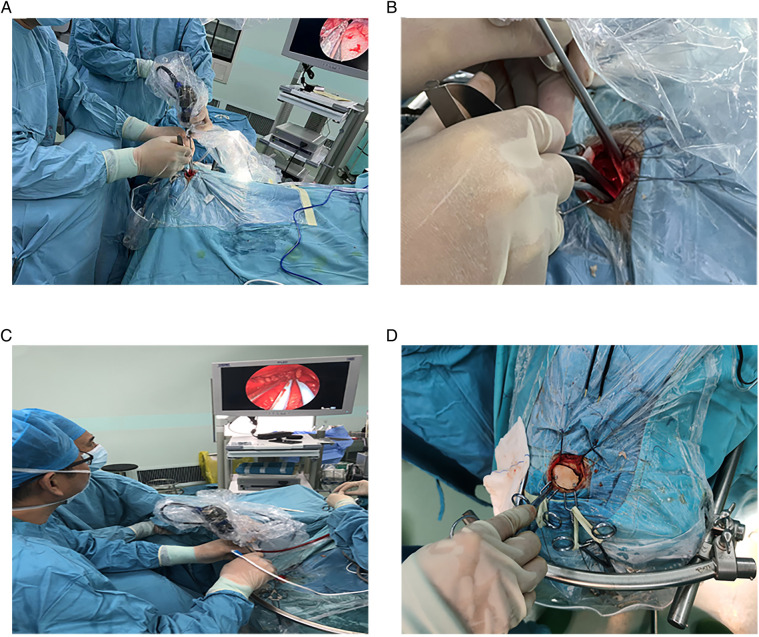
Subdural endoscopic operation. (**A–C**) Location of the endoscope, instrument, and operators. (**D**) Resetting of the bone flap.

General anesthesia was performed following orotracheal intubation. A patient was in the supine position, and the head was rotated 10°–30° to the opposite side of the approach to the eyebrow arch (i.e., if the procedure was performed on the right eyebrow arch, the head was turned to the left), and the head was tilted back 10°–15°. The DORO head brace (Germany) was used to pin the patient's head. The whole upper edge of the eyebrow (Figures 2A–C) was taken, and a skin incision was made. The skin and subcutaneous tissues were cut with a blade (an electric knife was not and should not be used in this step of the procedure). The upper edge was retracted upward using two “fishhook-morphous” retractors, and the lower edge was untied downward with sutures to untie the wound while avoiding the use of a spreader (Figure 2D). This method reduces the incidence of upper eyelid swelling after surgery. A burr drill was used to drill behind the anterior temporal line, and the small free bone flap was removed with a milling blade (Figure 2E). The anterior boundary was flattened against the superior margin of the supraorbital nerve foramen (Figure 2F). If the frontal sinus was open, the mucous membrane in the sinus cavity was first removed, followed by electric cauterization with an electric knife and repeated flushing with hydrogen peroxide and diluted iodophor. Subsequently, the inner plate of the frontal sinus was removed by drilling, and the outer plate was retained (Figures 2G,H). A gentamicin-containing gelatin sponge was filled and sealed with bone wax. In this study, there were three cases of frontal sinus opening, and there was no occurrence of cerebrospinal fluid leakage or intracranial infection after the operation. A U-shaped cut of the dura was made, and it was turned to the eye side and suspended. Intravenous administration of mannitol, hyperventilation, and release of cerebrospinal fluid from the lateral fissure (Figure 2I) were performed to reduce the intracranial pressure. The use of an indwelling lumbar cistern external drainage tube was unnecessary. In this operation, the assistant held the endoscope, and the operator performed the microoperation (i.e., one hand was holding the suction apparatus while the other hand was holding bipolar coagulation, microscissors, forceps, etc.). The distribution of surgical instruments was triangular, with the endoscope at the apex and the remaining instruments at the base points on both sides (Figures 3A,B). The endoscope holder was on the left side of the operator, the display screen of the endoscope was on the opposite side to the operator, the hand-washing nurse was on the right side of the patient, and the anesthesiologist was behind the operator (Figure 3C). A STORZ 30° rigid endoscope was used. The endoscope gradually penetrated the anterior skull base using the supraorbital margin as the fulcrum. The instruments operated by the surgeon were located on both sides of the endoscope. The endoscope and instruments simultaneously entered and exited, and the instruments were constantly within the field of vision at the front of the endoscope.

For tumor resection, the tumor basilar part was initially removed under endoscopy. For tumors growing toward the pituitary fossa, the direction of the endoscope was adjusted, and a bending curette was applied to remove the tumor. Subsequently, piecemeal resection was performed for the tumor body.

Finally, *in-situ* interlocking or artificial repair suturing of the dura mater was performed. The sutured area was sealed with biological glue, a free bone flap was fixed with two peptide chain fixation, bone foramen and bone suture were filled with bone cubic, subcutaneous alignment was achieved by suturing, and the wound was closed with intradermal suturing (Figure 3D).

### Statistical analysis

Data are expressed as the mean ± standard deviation of the normal distribution for continuous variables and frequency or percentage for categorical variables. All statistical analyses were performed using the SPSS 23.0 (IBM Corp., Armonk, NY, USA) software. A *P*-value of <0.05 denoted a statistically significant difference.

## Results

Nineteen patients (mean age: 56 ± 13 years; 73.7% females) underwent supraorbital craniotomy. The main preoperative symptoms comprised visual dysfunction (*n* = 12) and headache and dizziness (*n* = 6), while one case was inadvertently found by physical examination. Preoperative olfactory testing and analysis of hormone levels in venous blood were normal.

MRI plain scan, enhanced scan, and CTA were performed before surgery. In 12 cases, the tumor was closely related to the internal carotid artery and/or anterior cerebral traffic artery complex. Among them, two cases were partially wrapped, and three cases were completely wrapped

All patients were pathologically diagnosed with WHO grade I meningioma. According to Kuga et al. (10), tumors are classified into three types according to the imaging relationship between TSM and chiasma: type I, tumors with an intact optic apparatus; type II, tumors in which the optic chiasm is pushed superiorly by the tumor from the ventral aspect; and type III, tumors in which the optic chiasm is pushed posteriorly by the tumor from the rostral aspect. Among 19 cases, 3, 5, and 11 cases were of types I, II, and III, respectively. The TSM grading scale proposed by Magill et al. (11) was used: tumor diameter (<17 mm: 1 point; ≥17 mm: 2 points), optic canal invasion (≤3 mm: 0 point; invades one canal and is >3 mm: 1 point; invades two canals and is >3 mm: 2 points), and arterial encapsulation (<180°: 1 point; ≥180°: 2 points). The score distribution in these 19 cases is shown in Table 1.6 patients had surgery on the left side, 13 on the right side,The mean operation time was 2.61 ± 0.85 h, and the mean tumor size was 3.6 ± 2.5 cm

**Figure 4:**
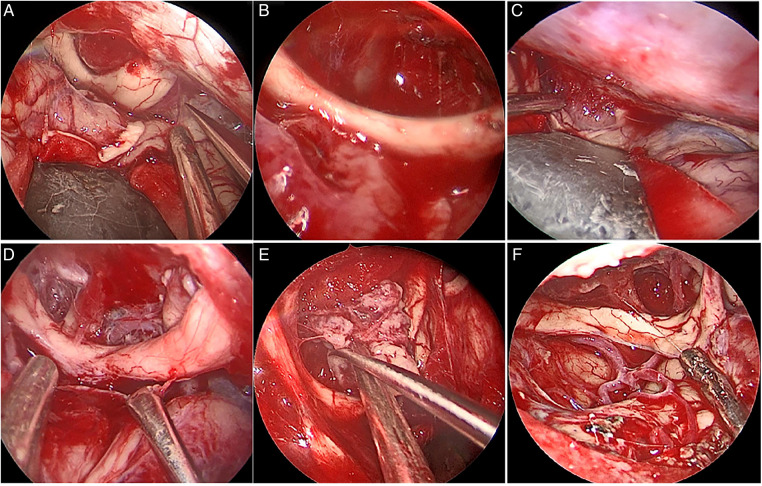
Intraoperative optic neural tube invasion (**A**) Under the optic nerve on the right side of the tumor and in the first space, push the optic nerve upward; (**B**) After total resection of the tumor (**C**) the main body of the tumor is located in the first space, and the right optic nerve is pushed upward and outward; (**D**) After total resection of the tumor.

The extent of resection was divided into gross total resection (GTR), near-total resection (NTR; 95%–99%), or subtotal resection (<95%) (12, 13). Of 19 patients in this group, 18 had GTR, and one had NTR. In this group, six cases had tumors that invaded the optic canal, two cases had tumors that pushed the optic nerve upward, and four cases had tumors that pushed the optic nerve laterally from the first space (Figure 4) (Table 2).

**Table 2:** Intraoperative conditions of TSM patients.

Extent of Resection	GTR	17 (94.4%)
	STR	1 (5.6%)
Kuga grade	I	3 (15.8%)
	II	5 (26.3%)
	III	11 (57.9%)
Magill Grading Scale	2	4 (21.1%)
	3	5 (26.3%)
	4	5 (26.3%)
	5	5 (26.3%)
Operation time,h	2.61 ± 0.85	
The length of the incision, cm	4.81 ± 0.15	
Size of bone flap, mm^2^	8.31 ± 0.65	
Optic canal invasion	yes	6 (31.6%)
	no	13 (68.4%)
Relationship with anterior cerebral communicating artery complex	Fully surrounded	3 (15.8%)
	Partially surrounded	2 (10.5%)
	Contact	12 (63.2%)

Postoperative visual function improved in 10/12 cases (83.3%), and dizziness was relieved in 5/6 cases (83.3%). Postoperative intracranial infection occurred in one case, which recovered after external drainage of the lumbar cistern and anti-infective treatment. Two cases of frontal lobe injury were discharged after conservative treatment. There was no occurrence of postoperative anosmia, eyelid ptosis, endocrine dysfunction, cerebrospinal fluid leakage, or death. There was no recurrence in total resection cases, no progress in subtotal resection cases, and no death cases during the follow-up of 7 months (Table 3).

**Table 3:** Postoperative conditions of TSM patients.

Postoperative Complication	Intracranial infection	1 (5.6%)
	The frontal lobe damage	2 (10.5%)
	New vision loss	0
	Stroke	0
	Hematoma	0
	CSF leak	0
	New hypopituitarism	0
	New anosmia	0
Visual outcome	Improved	10 (83.3%)
	Unchanged	2 (16.7%)
	Worsened	0
headache outcome	Improved	5 (83.3%)
	Unchanged	1 (16.7%)
	Worsened	0
Recurrence/Mortality		0

## Discussion

TSM accounts for 5%–10% of all meningiomas (14–16). These tumors originate from the planum sphenoidale, optic sulcus, and sphenoid margin and can extend to nearby areas, such as the sphenoid plateau, saddle area, posterior clinoid process, and cavernous sinus (16). Owing to the complex anatomical structure of this region, which includes important blood vessels and nerves (optic nerve, optic chiasm, internal carotid artery, anterior cerebral artery complex, cavernous sinus, pituitary stalk, etc.), it is difficult to achieve Simpson I level TSM resection (14, 15).

Surgical resection, the preferred treatment for TSM, can be divided into TCA and nasal approaches according to the employed surgical method. The former includes the pterional approach, unilateral subfrontal approach, and bilateral interfrontal hemispheric approach and has been established as the standard method for surgical TSM resection for decades (2). In recent decades, significant progress has been achieved in EEA for TSM treatment with the rapid development of endoscopic technology (4). Its main advantages are a limited disturbance to brain tissue and optic nerve optic chiasma, effective treatment of the tumor base, and eventual removal of the involved dura mater and skull, rendering Simpson I resection possible. Furthermore, limitations of the EEA for TSM treatment also exist. First, the indications are limited; for example, the EEA is not applicable to vascular wrapping and laterally growing tumors or those with a size of >2–2.5 cm (17). Second, skull base reconstruction and cerebrospinal fluid (CSF) rhinorrhea are important problems limiting the development of EEA (18, 19). Studies have suggested that the incidence of CSF rhinorrhea after the treatment with TSM by EEA is 23%–40% (20). Although the technique for skull base reconstruction has improved, the incidence of CSF rhinorrhea remains at 5%–10%. This rate is significantly higher than that associated with transcranial surgery. Finally, the conchal sphenoid sinus and operative side accessory sinus inflammation are also key factors affecting the efficacy of EEA (7). There is an ongoing debate regarding the most appropriate surgical approach (i.e., TCA or EEA) in this setting (18). In a meta-analysis, Yang et al. (4) compared TCA and EEA regarding the tumor resection rate, recurrence rate, vision improvement, and CSF leakage. They concluded that there was no difference in tumor resection and recurrence rates. In their study, EEA was superior to TCA in terms of vision improvement; however, the rate of CSF leakage has been higher with EEA vs. TCA. Studies have suggested that there is no difference in postoperative recurrence rate between the two methods (21). Notably, there is also disagreement regarding the selection of EEA and endoscopic TCA (22, 23). Recently, a more effective approach has been proposed, namely, the supraorbital approach (6, 24, 25). Linsler et al. (26) suggested using the supraorbital approach for the treatment of larger TSM tumors growing to the far lateral side of the saddle region or those wrapped with blood vessels. Using a microscope, endoscope, and neural navigation, Arnaout et al. (9) performed eight operations through the supraorbital approach on four cadaver heads. They compared the visibility and accessibility of the anterior and middle cranial fossa regions. The results have demonstrated that both endoscopic and microscopic images provided the surgeon with nearly identical visibility and accessibility and that the supraorbital keyhole approach was preferable to endoscopy alone. Berhouma et al. (8) also demonstrated the feasibility of endoscopic resection of anterior middle skull base lesions through the supraorbital keyhole approach.

**Figure 5:**
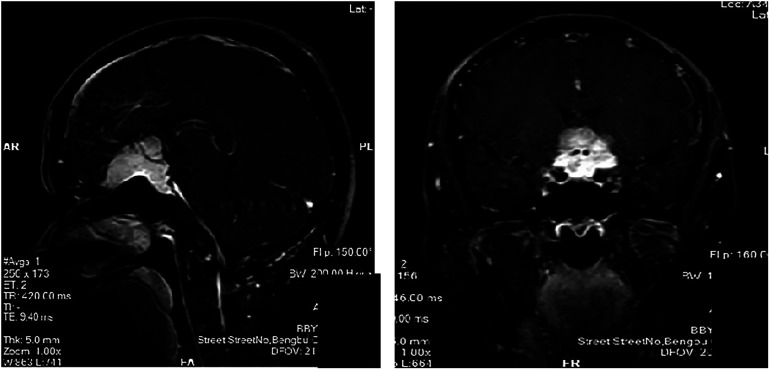
The tumor completely surrounded bilateral anterior cerebral arteries.

In this study of 19 cases of TSM, the tumors were removed by pure neuroendoscopy *via* the eyebrow arch approach, achieving good clinical results. We selected the extent of resection as the standard for tumor resection instead of the Simpson score. This decision was based on studies that suggested the absence of difference in postoperative recurrence rate and recurrence-free survival rate between Simpson grade I–III meningiomas (13) and that total tumor resection could lead to good outcomes. Oya et al. (24) have also confirmed no correlation between the resection of Simpson grade I–III WHO meningioma and recurrence-free survival. According to the tumor grades of Kuga D (10) and Magill (11), all cases in this group were covered. According to EOR grading, 18 of 19 patients in this group had GTR, and 1 had NTR. In a case of the tumor with preoperative Kuga grade III, Magill score of 5, and tumor size of 4 cm  ×  5 cm × 5 cm, intraoperatively, it was closely adhered to the right anterior cerebral artery and the initial part of the middle cerebral artery without clear boundaries and could not be removed even by using a microscope (Figure 4). Bernat et al. (21) indicated that bone dysplasia, ICA, invasion of the anterior cerebral artery and middle cerebral artery, and maximum dural tail sign in the transverse section are all considered independent factors for incomplete resection of anterior skull base tumor. No serious complications, such as second operation, cerebrospinal fluid leakage, or death, occurred. There was one case of postoperative intracranial infection in which the frontal sinus was not opened. Considering that the cause of infection might be related to the large tumor and long operation time (4 h 23 min), it has been reported in the literature that the operation time of >4 h (27) and a large benign tumor of the skull base (28) are the risk factors of intracranial infection after craniotomy. Postoperative visual function was improved in 6/7 cases (85.7%). Additionally, there was one case where the postoperative visual function was not improved, and the patient was blind in his right eye before surgery. It has been pointed out in the literature (1) that postoperative improvement might be lower in patients with poor preoperative visual acuity. In this group, postoperative follow-up lasted at least 3 months, and no recurrence was found. However, due to the short follow-up, postoperative recurrence could not be accurately reflected, and further follow-up is required. It has been reported that the optimal follow-up time after TSM is 5–10 years (2). In this group, six cases had tumors that invaded the optic nerve canal, two cases had tumors that pushed the optic nerve upward (Figures 5A–D), and four cases had tumors that pushed the optic nerve outwardly from the first space (Figures 5E–F). During the operation, the optic nerve canal was not opened by 30°-endoscope and angular curettage.

### Summary of technical requirements and surgical techniques

Robinow et al. (4) indicated that the supraorbital approach is most suitable for anterior skull base tumors, such as TSM, and can replace the previous pterotomy. Louis et al. (6) pointed out that there were four blind areas in the microscopical approach through the brow arch: the anterior part of the olfactory groove, the sellar bottom, the inferior part of the ipsilateral optic nerve, and the anterior part of the middle cranial fossa under the sphenoid crest. Arnaout (8), such as using the microscope and endoscope and neural navigation, has performed on the eight to four cadaver head anatomy of the supraorbital superciliary arch into the road, the front, middle cranial fossa region of visibility and accessibility, conclusion endoscopy and microscopic image can provide the surgeon with almost the same visibility and accessible area so the orbital lock hole can adopt pure endoscopic approach into the road. Berhouma et al. (7) also demonstrated that total endoscopic resection of anterior middle skull base lesions through eyebrow arch keyhole is feasible. Therefore, we believe that the fully endoscopic supraorbital trans-eyebrow keyhole approach is feasible.

Endoscopic TSM resection through the supraorbital keyhole approach offers the following advantages. First, there are four blind areas in the supraorbital approach using a microscope: the anterior part of the olfactory groove, the sellar floor, the part inferior to the ipsilateral optic nerve, and the anterior part of the middle cranial fossa below the sphenoid crest (7). This problem can be overcome through the use of an angle lens in neuroendoscopy. Second, most scholars use neuroendoscopy only as the auxiliary lighting of the microscope. Therefore, two sets of equipment need to be prepared, and continuous switching between the two sets is necessary during the operation. The use of total endoscopy is effective in avoiding this process. Giammattei et al. (29) showed that EEA could be a better approach in cases of tumors that tend to extend deep in the sella turcica, suggesting evaluating the angle from the frontobasal line to the sella. These authors also found that optic canal invasion is a good indication of EEA due to the possibility of performing an early decompression of the medial part of the canal.

Due to the saddle nodules, meningioma often invades the optic canal and optic nerve or pushes it laterally, and the optic microscope below the eyebrow bending into the road is one of the four blind areas (7). The other three are the front of the olfactory groove, the bottom of the saddle, and the sphenoid ridge of the cranial fossa in the front; thus, surgeons often need to grind the optic nerve tube wall to cut the tumor. In this study, we used 30° endoscopy and angled curettage to remove residual tumors without opening the optic canal wall. However, due to the small number of cases in this study, tumor invasion of the optic canal was not too serious; hence, a large number of cases needs to be further summarized.

Below, we outline our experience during the fully endoscopic supraorbital trans-eyebrow keyhole approach. 1. When the surgery is performed, the first choice is the side with severe visual impairment before surgery, followed by the side with frontal sinus occlusion or smaller; when the two conditions are similar, the side of the nondominant hemisphere is selected. 2. Open frontal sinus will increase the rate of postoperative infection rate; thus, appropriate disinfection measures and simultaneous strict closure of the frontal sinus are essential. 3. In addition to conventional epidural grinding of the anterior skull base bone ridge, the inner plate of the frontal sinus needs to be burnished to provide sufficient operating space and a stable fulcrum for subsequent endoscopic operation. 4. The supraorbital nerve must be preserved, and damage should be avoided. 5. We used the assistant to handle the endoscope with both hands for microscopic operation. This method can adjust the depth and angle of the endoscope as the surgeon operates, and the assistant can achieve a better endoscope flexibly by handling the endoscope with both hands compared to the mechanical arm (30, 31). Moreover, unlike the fixation arm, endoscopy can achieve “dynamic magnification” (32, 33). The fulcrum of the assistant handling the endoscope. Usually, TSM tumors are located in deep sites; hence, a stable and reliable fulcrum is needed after in-depth endoscopy. In the absence of a fulcrum, the assistant handling the endoscope is prone to shaking and fatigue. There are two fulcrums: the endoscopic fulcrum (i.e., the superior orbital margin of the bone window) and the arm fulcrum (i.e., the right hand dragging the left elbow joint). 6. Endoscopy has a proximal visual blind area; hence, endoscopy and other surgical instruments (suction, microscissors, etc.) into the easy loss after normal blood vessels or nerves, causing serious complications (8). Based on our experience, the assistant holds the endoscope, places it in the middle, and gradually penetrates along the anterior skull base. The surgeon enters through both sides of the endoscope, with both hands holding instruments. At the same time, the assistant holding the endoscope should ensure that the instrument is located at the front end of the endoscope and simultaneous entry and exit. In every operation, the surgeon should ensure that the instrument is under the vision field of the endoscope.

### Limitations and generalizability

The main limitation of this study was its short follow-up period. The shortest follow-up period was only 3 months, which does not accurately assess the risk of postoperative recurrence. Furthermore, the number of cases included in this study was relatively small. Therefore, further multicenter studies with larger sample sizes are warranted to validate the presented findings.

## Conclusion

Neuroendoscopic TSM resection through the supraorbital approach is safe and feasible. It can be used to explore angles that cannot be seen under a microscope and show the true value of endoscopy technology. However, it requires a high level of endoscopic competence by the surgeon and assistant, which is acquired through long-term and repeated practice. Once this technique is mastered, it becomes a simple and time-saving surgical option.

## Data Availability

The raw data supporting the conclusions of this article will be made available by the authors, without undue reservation.
